# Changes in Mitochondrial Transcriptional Rhythms and Depression-like Behavior in the Hippocampus of IL-33-Overexpressing Mice

**DOI:** 10.3390/ijms26051895

**Published:** 2025-02-22

**Authors:** Yang Li, Weinan Gao, Lin Jiao, Delu Dong, Liankun Sun, Yanan Liu, Luyan Shen

**Affiliations:** 1 Key Laboratory of Pathobiology, Department of Pathophysiology, College of Basic Medical Sciences, Jilin University, Changchun 130021, China; liyang_2015@jlu.edu.cn (Y.L.); gaown23@mails.jlu.edu.cn (W.G.); jiao000lin@163.com (L.J.); dongdl@jlu.edu.cn (D.D.); sunlk@jlu.edu.cn (L.S.); 2 Department of Physiology, College of Basic Medical Sciences, Jilin University, Changchun 130021, China; 3 Yibin Research Institute of Jilin University, Yibin 644000, China

**Keywords:** neuroinflammation, circadian rhythms, depression-like behavior, mitochondria, RNA-seq

## Abstract

Neuroinflammation is involved in the development of depression and may induce depression-like behaviors by affecting metabolism through interactions with circadian rhythms. As the hub of metabolism, mitochondria are regulated by various types of metabolism and release signals that regulate cellular functions. In this study, we performed transcriptomic analysis of the hippocampus of IL-33-overexpressing mice to provide new ideas to explore the pathogenesis of inflammation-mediated depression at the transcriptional level. Male C57BL/6J mice and IL-33-overexpressing mice were subjected to behavioral tests. The hippocampus was extracted during the light or dark period, and differential gene expression analysis was conducted using RNA sequencing. Differential gene enrichment analysis was performed, as well as multilayered analysis of mitochondrial transcriptional rhythms by integrating the regulatory networks and Mito 3.0 database. The results were further verified using RT-qPCR. IL-33-overexpressing mice exhibited depressive behaviors associated with rhythmic disorders and shortened circadian cycles. Differential KEGG (Kyoto Encyclopedia of Genes and Genomes) enrichment analysis showed that the top 20 pathways with the lowest *p*-values included mood-related, immune-related, and circadian rhythm-related pathways. Differential gene GO (Gene Ontology) enrichment analysis showed that 20 of the top 30 pathways with the lowest *p*-values were related to metabolism. Transcriptome data from IL-33-overexpressing mice showed that the mitochondrial-encoded subunit of the oxidative respiratory complex showed predominantly increased expression during the light period. Metabolic disorders and disrupted mitochondrial transcriptional rhythm were also observed. Weighted gene correlation network analysis showed that the circadian cycle is associated with depression-like behavior disorders. Network analysis showed that circadian-related genes were enriched in mitochondrial pathways related to metabolism and oxidative phosphorylation. Multilayer analysis of mitochondrial transcriptional rhythms using the mitochondrial database Mito 3.0 revealed that mitochondrial dynamics and surveillance pathways were the most enriched. The depressive behavior in mice caused by long-term IL-33 stimulation may be related to changes in the transcriptional rhythms of metabolism-related genes and the interaction between mitochondria and clock genes. This suggests that mitochondrial transcriptional rhythms are central to the pathogenesis of microinflammation-induced depression, further supporting the potential of mitochondria as a target for the prevention and treatment of depression.

## 1. Introduction

The prevalence of mood-related disorders, such as depression, is increasing annually as work and life stresses increase. Circadian rhythm disruption is a potentially important factor affecting the efficiency of depression medication and the prevalence of recurrent episodes. Recent findings indicate that stress can cause neuroinflammation, which in turn disrupts circadian rhythms [1]. The findings suggest that the pathogenesis of mood-related disorders such as depression can be further clarified from the perspective of neuroinflammation leading to the disruption of circadian rhythms.

Research has demonstrated that approximately 50% of the genes responsible for encoding proteins in mammals exhibit circadian transcriptional rhythms, predominantly in an organ-specific manner [2], suggesting that rhythmic abnormalities in depression-associated brain regions may facilitate significant progress in the elucidation of mood-related disorders such as depression. The hippocampal volume is known to shrink in depressed patients under chronic stress, anxiety, and other stressful stimuli. Furthermore, the expression of hippocampal rhythm gene mRNAs and proteins exhibits circadian oscillations. However, we do not yet understand how these changes influence mood [3]. A Cell Metab study utilizing the technique of chromatin immunoprecipitation sequencing of liver rhythm genes found that the target gene functions of rhythm genes are involved in processes such as the oxidative phosphorylation complex, tricarboxylic acid (TCA) cycle, biosynthesis, protein synthesis, kinetics, β-oxidation, metabolite transporters, glucose metabolism, and autophagy [4]. Mitochondria may act as an early receptor for circadian rhythm, and their abnormal function is closely related to circadian rhythm disorders.

A study highlighted that microinflammation (also termed hypoinflammation) exhibits biological mechanisms analogous to the depressive state induced by chronic stress [5]. Non-steroidal anti-inflammatory drugs reduce the state of major depression more than placebo [6], and anti-inflammatory drugs are effective in reducing mania and depression in people with bipolar disorder [7]. Indeed, it is now believed that inflammation regulates circadian rhythms, primarily through the regulation of clock genes by inflammatory factors [8,9,10]. Interleukin (IL)-33, which is highly expressed in the central nervous system (CNS), is upregulated and released in response to inflammatory stimuli after injury. IL-33 acts as an alarm and contributes to tissue homeostasis and response to environmental stresses. In addition, it serves as an important inflammatory factor in the context of neurodegenerative diseases and cognitive impairment. Although it is currently believed that inflammation can influence circadian rhythms, studies on the role of IL-33 as an alarm molecule in rhythms are still limited. IL-33-mediated mast cell activation exhibits circadian rhythmicity [11], suggesting that IL-33, an important factor in responding to the environment, may have an impact on circadian rhythms.

Recent studies have proposed that the circadian rhythm of mitochondria is characterized by mitochondrial dynamics and cycles of fission and fusion; molecular content, that is, the expression of mitochondria-associated genes (encoded by the nucleus and mitochondria), as well as at the protein level (e.g., key mitochondrial enzymes) and metabolites; and major mitochondrial functions, such as oxidative phosphorylation (oxygen consumption rate assay) [12,13]. Changes in mitochondrial DNA, including the expression levels of mitochondrial genes and mutations, have been observed in patients with depression, suggesting changes in mitochondrial function and alterations in normal cellular metabolism. Impaired mitochondrial function reduces intracellular ATP production, which may lead to fatigue symptoms and lack of energy experienced by depressed patients [14]. Previous studies have shown that there is a crosstalk between circadian rhythms and various mitochondrial processes, including fission-fusion kinetics and mitochondrial energetics such as oxidative phosphorylation and ATP production, all of which are strongly regulated by the clock [15]. Mitochondrial function under the regulation of clock genes exhibits circadian changes, while mitochondria may affect transcription through structural changes, deacetylation mechanisms, and ATP and reactive oxygen species (ROSs) production [16]. This may result in mitochondria-centered transcriptional rhythms, which in turn reverse signaling to affect circadian rhythms. In recent years, it has been suggested that IL-33 is a key regulator of mitochondrial metabolism and mitochondrial respiration [17,18] and also involved in the regulation of mitochondrial autophagy [19]. The effect of IL-33 on mitochondrial circadian rhythms may be an important mechanism in the development of depression. Studying mitochondrial transcriptional rhythms may be important for understanding the development of circadian rhythm disorders. In this study, we used transcriptomics to explore how circadian rhythms may interact with depression under microinflammation. Given the plethora of big data analysis methods available, each with its own unique algorithms and tendencies, we employed multiple analysis methods to validate the generalizability of our findings. This involved the combination of WGCNA analysis with datasets available online. This research is characterized by making full use of multiple approaches to validate and analyze and combining the existing theories and research bases in the laboratory, which is more conducive to the discovery of new mechanisms and theories. This paper focuses on analyzing data from existing databases and IL-33-overexpressing mice, using multiple big data analytics methods, to gain insights into mitochondrial changes and potential mechanisms in circadian rhythm disruption and depression-like behaviors due to microinflammation.

In this study, we employed IL-33-overexpressing mice to detect and observe circadian rhythmic behavioral changes through anxiety- and depression-like behaviors, combined with transcriptomics large-scale analysis. The aim of this research was to provide a basis for revealing the possible mechanisms of transcriptomic and mitochondrial circadian rhythmic changes under inflammatory stress on the psychological state and social behaviors of humans. Additionally, this study sought to explore the effects of chronomedicine and mitochondrial medicine on depression-like behaviors.

## 2. Results

### 2.1. Depression- and Anxiety-like Behavioral Changes in IL-33-Overexpressing Mice

**Figure 1:**
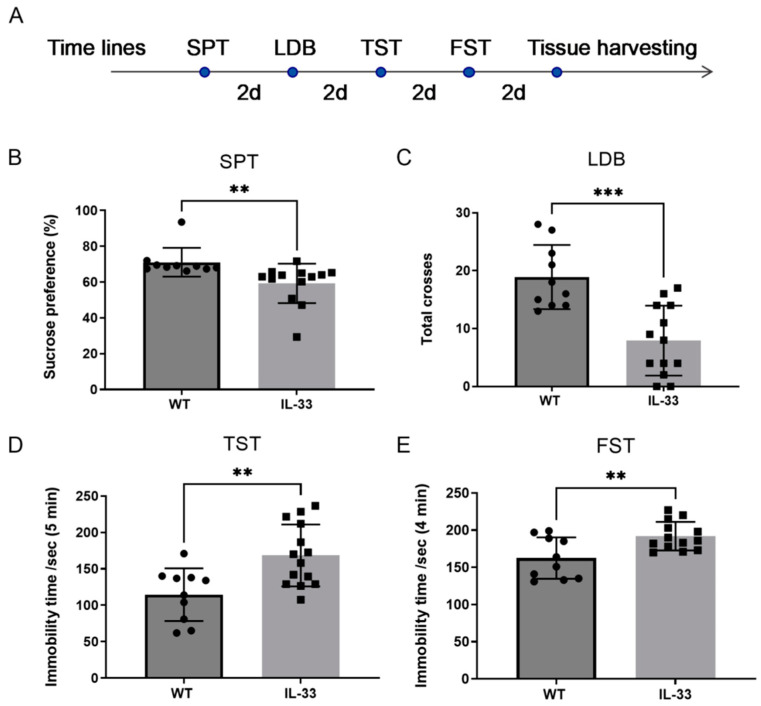
Behavioral changes in IL-33-overexpressing mice. (**A**) Experimental design of behavioral studies. (**B**) Rate of sugar–water preference in the sugar–water preference experiment. (**C**) Number of shuttles in the black-and-white box experiment. (**D**) Immobility time in the tail-hanging experiment. (**E**) Immobility time in the forced-swimming experiment. There were 10–14 mice in each group, statistically different from WT mice (** *p* < 0.01, and *** *p* < 0.001).

To determine whether the mice lacked pleasure, in the SPT test, the sugar–water preference rate of the WT mice was 71.06 ± 8.05, while that of IL-33-overexpressing mice was significantly lower at 59.31 ± 11.00 (** *p* < 0.01, Figure 1B). The number of crosses between the light and dark boxes in 5 min was 7.92 ± 6.05 in IL-33-overexpressing mice and 18.90 ± 5.55 in WT mice, and the number of shuttles in IL-33-overexpressing mice was significantly lower than that in the WT group (*** *p* < 0.001, Figure 1C). In the TST (Figure 1D), the immobility time in 5 min in WT mice was 114.6 ± 36.09 s, which was significantly lower than that of IL-33-overexpressing mice, which was 168.5 ± 42.54 s (** *p* < 0.01). In the FST, the immobility time of IL-33-overexpressing mice within 4 min was 192 ± 19.14 s, which was significantly higher than the immobility time of WT mice, which was 162.5 ± 27.78 s (** *p* < 0.01; middle panel Figure 1E). The IL-33-overexpressing mice showed some anxiety- and depression-like behaviors.

### 2.2. Changes in Activity Rhythms in IL-33-Overexpressing Mice

**Figure 2:**
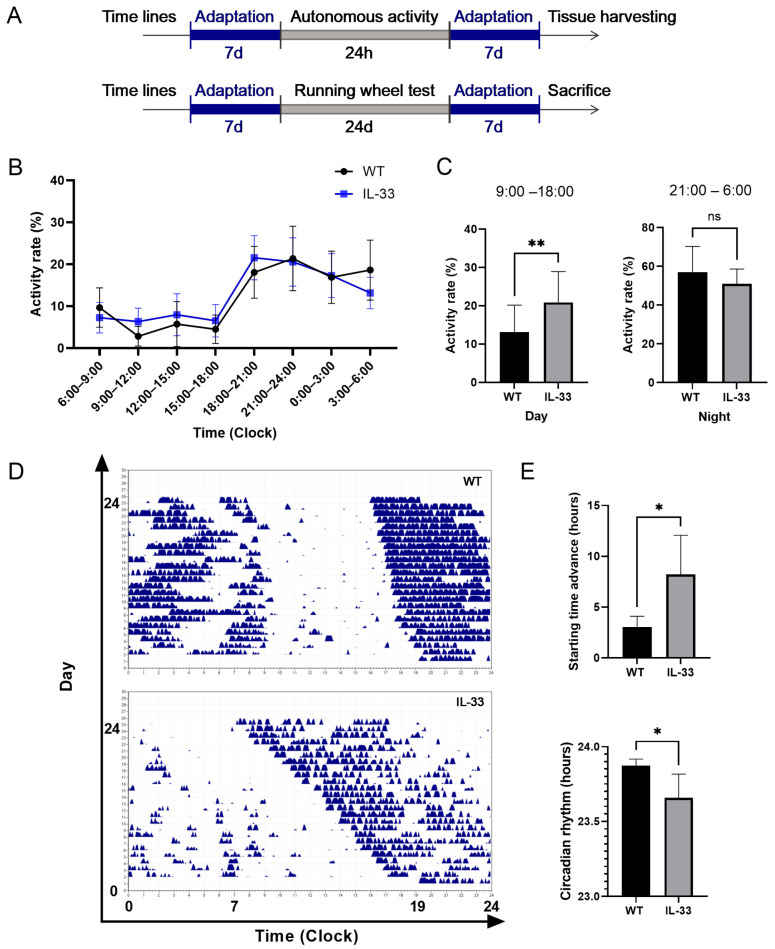
Behavioral changes related to circadian rhythms in WT and IL-33-overexpressing mice. (**A**) Experimental design of activity rhythm studies. (**B**) Percentage of autonomic activity in different time periods during 24 h in both groups. (**C**) Percentage of activity rate in partial light and dark periods. (**D**) Graph of the active running wheel results. (**E**) Starting time advance and circadian rhythm of WT and IL-33-overexpressing mice. There were 4–5 mice in each group, * *p* < 0.05 and ** *p* < 0.01, statistically different compared with the WT group *p* > 0.05.

A small animal autonomous activity recorder was used to detect activities of mice in different time segments over 24 h, which was then divided by the total number of activities over 24 h to obtain the activity rate (%). The day was divided into eight time segments, with each 3 h serving as a time segment, the first of which started at 6:00–9:00 a.m. The results show that both WT mice and IL-33-overexpressing mice (Figure 2B) exhibit lower activity in the light period than the activity rate in the dark phase. The results of two-way ANOVA (Figure 2D) showed no significant difference in activity rates between the two groups of mice, but both showed time-dependent variability, with an interaction between IL-33-overexpression and temporal aspects (*p* = 0.002). In the statistics of the time periods when the peak, trough, and onset of activity occurred in mice, it was found that the trough time period of activity in WT mice and IL-33-overexpressing mice was from 9:00 to 12:00, and the onset time period was from 18:00 to 21:00 (Figure 2C). The peak time period differed from 21:00 to 24:00 in WT mice, while that in IL-33-overexpressing mice was from 18:00 to 21:00, showing a forward shift in the peak time period of activity and rhythmic changes in activity. WT and IL-33-overexpressing mice were free to move in the standard environment of L:D (12 h:12 h), and their running wheel time was stable for a period of 24 h. After removing the effect of light and dark environments, the mice were observed for the changes in their own circadian rhythms. Figure 2D shows the daily activity graphs of the mice for 24 days, and the onset of the autonomous running wheel of the WT mice was advanced by 3.05 ± 1.05 h after 24 days of continuous dark environment; the average self-circadian rhythm was 23.87 ± 0.13 h. In IL-33-overexpressing mice, the circadian rhythm was advanced by 6.58 ± 4.56 h after 24 days (*p* < 0.05) compared with WT mice (Figure 2E), and the average self-circadian rhythm was 23.73 ± 0.26 h (*p* < 0.05) compared with WT mice (Figure 2E). These results suggest that IL-33-overexpressing mice have a shortened circadian cycle rhythm compared with WT mice.

### 2.3. Enrichment Analysis of Differentially Expressed Genes

**Figure 3:**
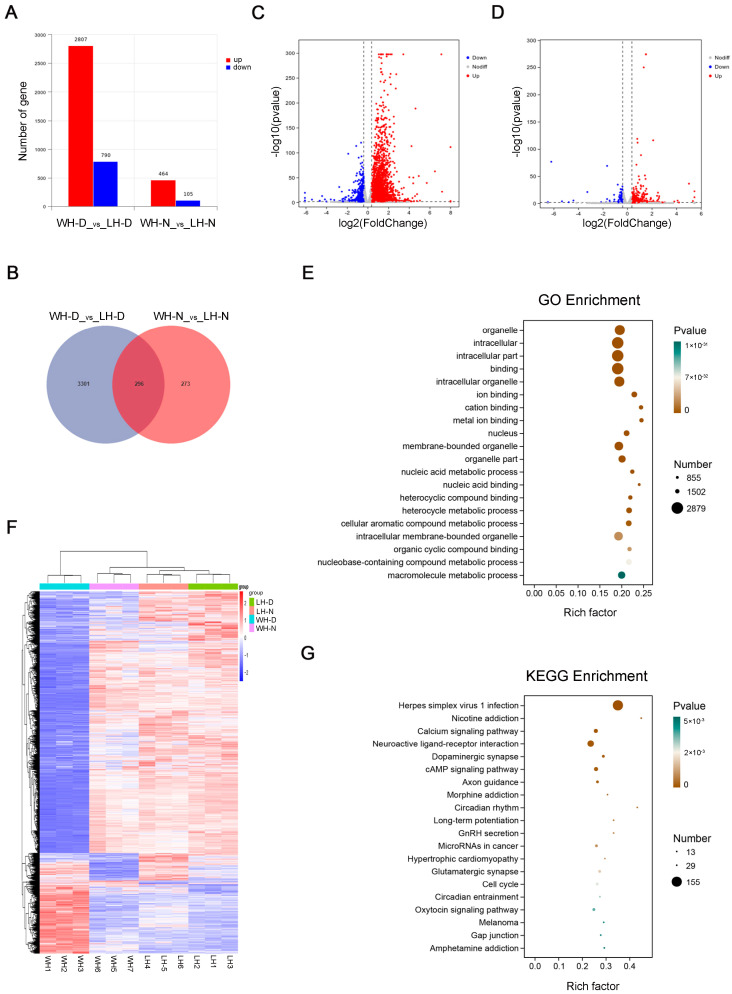
Day- and night-time differential gene analysis. (**A**) Bar chart of the differential gene number. (**B**) Differential gene Wayne plots. (**C**,**D**) Differential gene volcano plots of day and night, respectively. (**E**) Daytime differential gene GO enrichment analysis. (**F**) Gene expression heatmaps. (**G**) Daytime differential gene the KEGG enrichment analysis. WH-D: hippocampal tissue from wild mice during the day; LH-D: hippocampal tissue from IL-33-overexpressing mice during the day; WH-N: hippocampal tissue from wild mice during the night; LH-N: hippocampal tissue from IL-33-overexpressing mice during the night.

To investigate how IL-33 gene overexpression leads to exhibiting depressive tendency behaviors, as well as the cause of rhythmic changes in mice, we next detected gene expression changes in the hippocampal tissues of WT and IL-33-overexpressing mice during two time periods, 9:00 to 11:00 a.m. in the morning (light period) and 21:00 to 23:00 p.m. in the dark period, using transcriptomics technology and enriched the analysis of all differentially expressed genes. In Figure 3A, the two bar graphs on the left panel show the differential gene status in hippocampal tissues during the daytime in WT mice and IL-33-overexpressing mice, with the red bars showing the number of genes with increased expression of 2807 genes and the blue bars showing the number of genes with decreased expression of 790 genes. The two bars on the right represent hippocampal differential gene profiles in the two groups of mice at night, with 464 genes exhibiting elevated expression and 105 genes exhibiting decreased expression. Figure 3B shows a Wayne plot of the differentially expressed genes, showing 296 genes that varied during both day and night. Figure 3C,D show volcano plots, where red dots express elevated genes, blue dots express reduced genes, vertical coordinates are negative logarithms with a base of 10 for *p*-values, and larger vertical coordinates represent smaller *p*-values. The horizontal coordinate is the logarithm of the differential expression multiplicity with 2 as the base, and a larger absolute value of the horizontal coordinate represents a larger change in the expression multiplicity. From the volcano plot, it can be seen that the genes with elevated expression during the daytime are larger than those with decreased expression, the trend of change at night is the same as that during the daytime, and the number of differentially expressed genes at night is smaller than that during the daytime. Figure 3F plot is a heatmap of the differential expression of genes. The graph is divided into the following four major parts: daytime gene expression in WT mice (blue bar) and nighttime gene expression in WT mice (purple bar). Most of the gene expression in these two sections shows an opposite trend, suggesting circadian changes in the expression of some genes. The orange bar corresponds to gene expression at night in IL-33-overexpressing mice, and the green bar corresponds to gene expression during the day in IL-33-overexpressing mice. The circadian changes in gene expression in IL-33-overexpressing mice were not as obvious as those in WT mice, and the circadian expression oscillations of many genes disappeared. This indicates that circadian rhythmic changes in gene transcription, which appear in WT mice, are ameliorated in IL-33-overexpressing mice. Figure 3E shows the GO enrichment analysis factor plot of differential genes, and the top 20 pathways with the smallest *p*-values are shown, among which the pathways in the biological process (BP) are nucleic acid metabolic process, heterocycle metabolic process, cellular aromatic compound metabolic process, and nucleobase-containing compound metabolic process, all of which are metabolism-related. Twenty of the top 30 pathways with the smallest *p*-values of pathway change in BP are metabolism-related. Figure 3G shows the KEGG enrichment analysis factor plot of differentially expressed genes, which lists the top 20 pathways with the smallest *p*-values, including immune-related pathways such as “Herpes simplex virus 1 infection and Neuroactive ligand-receptor interaction”. The pathways related to mood were “nicotine addiction, calcium signaling pathway, dopaminergic synapse, long-term potentiation, and glutamatergic synapse.” Circadian rhythm and circadian entrainment were pathways related to circadian rhythm. These findings suggest that the anxiety- and depression-like behavior of IL-33-overexpressing mice may be related to metabolism, which is jointly regulated by immune, emotional, and circadian pathways.

Therefore, we focused on the changes in the expression of clock genes and mitochondrial oxidative respiratory complex genes in IL-33-overexpressing mice. The expression changes in major clock genes are presented in Table 1. The clock genes with increased expression in the daytime were Arntl2, Clock, and Rorα, while those with decreased expression were Cry1, Nr1d1, Npas2, and Dbp. The genes with increased nighttime expression were Arntl2, whereas the other genes exhibited no significant change. Table 2 lists the mRNA expression changes of the mitochondrial oxidative respiratory complex subunits. We set the multiplicity of increase or decrease to greater than 1.3, while Padj < 0.05 was considered significant for expression. The results showed that the mRNAs with increased expression in the daytime hippocampus were mt-Nd1, mt-Nd2, mt-Nd4, mt-Nd4l, mt-Nd5, mt-Nd6, and Ndufb2 in complex I; mt-Cytb in complex III; mt-Co1, mt-Co2, and mt-Co3 in complex IV; and mt-Atp6, Atp5l in complex V, most of which are encoded by mitochondria. Expression of these subunits differed significantly in the daytime hippocampus. The subunits with decreased expression were Uqcrc1 in complex III, Cox6a2 in complex IV, and Atp5b in complex V, all nuclear-encoded subunits. Night hippocampal transcriptomics revealed that the subunits with increased expression were Ndufab1 in complex I and Cox6b2 in complex IV. We found that the genes with increased expression during the daytime were mostly mitochondria-encoded, the genes with decreased expression were nuclear-encoded, and the genes with increased expression at night were nuclear-encoded.

### 2.4. WGCNA

**Figure 4:**
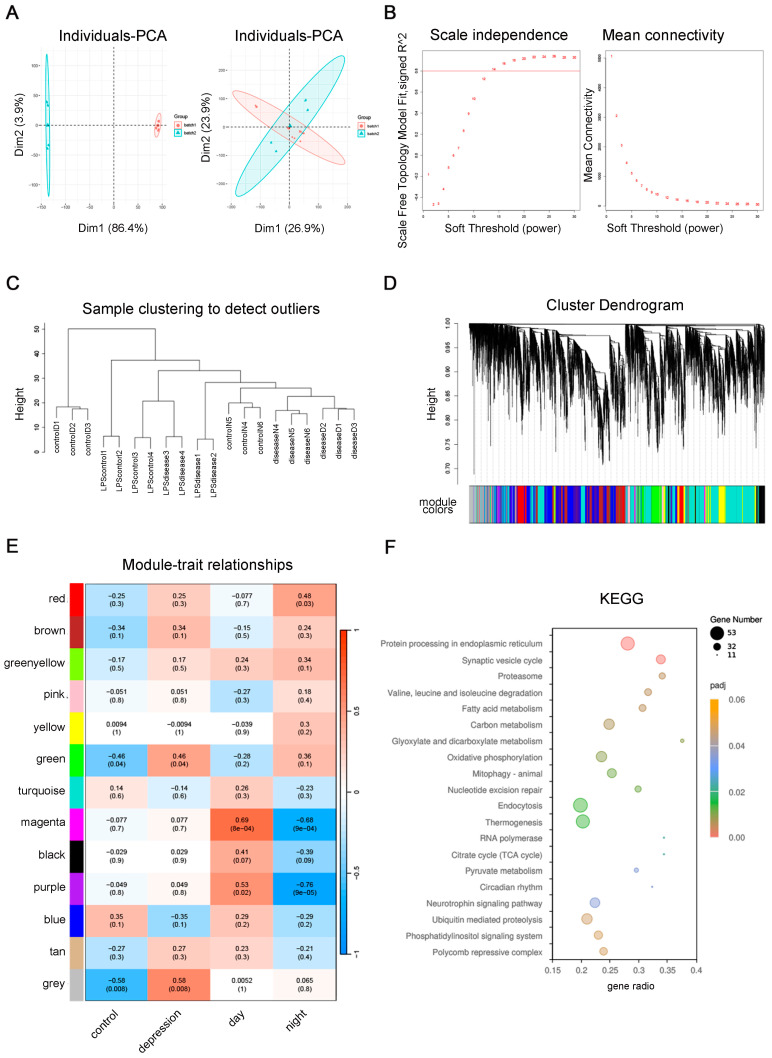
WGCNA validation of differential genes in LPS-induced neuroinflammation and IL-33-overexpressing mice. (**A**) Before and after plots of the batch correction of the data. (**B**) Soft threshold set to 16 by the machine model. (**C**) Module plot. (**D**) Dendrogram. (**E**) Module-trait correlation analysis. (**F**) KEGG analysis results of the genes combined within the green and blue modules.

To verify whether the results of the differential analysis of IL-33-overexpressing mice are generalizable, we used the GSE181285 database to obtain transcriptomics data for the lipopolysaccharide (LPS)-induced neuroinflammation model. After correcting the batches of data for the two groups, the soft threshold was set to 16 according to the machine model; type was set to sign, cor was set to bicor, power was set to 16, and merge module coefficient was set to 0.3, resulting in a total of 13 modules (Figure 4A–E). The WT mice in this experiment were combined with the mice in the saline control group in GSE181285 as the control group and were combined with the IL-33-overexpression group, the LPS model group, and the IL-33-overexpression and LPS model group as the model group. The daytime WT and IL-33-overexpression data were taken as Day, and the nighttime WT and IL-33-overexpression data were taken together as Night, and the modules were taken for trait correlation analysis. After comparing the correlation coefficients and *p*-values, the green and blue modules were taken for subsequent analysis. The green module was negatively correlated with the control and day groups and symptomatically correlated with the IL-33 group, the model group, and the night group; the blue module was positively correlated with the control group, and the daytime traits were negatively correlated with the IL-33-overexpression group, the model group, and the night group. KEGG analysis of the genes within the green and blue modules combined showed significant enrichment of genes within the modules in protein processing in the endoplasmic reticulum, synaptic vesicle cycle, proteasome, valine, leucine, and isoleucine degradation, fatty acid metabolism, carbon metabolism, glyoxylate and discarboxylate metabolism, oxidative phosphorylation, and mitophagy (Figure 4F). It has been suggested that genes associated with IL-33-overexpression, LPS-induced neuroinflammatory models, and circadian traits are enriched in pathways related to mitochondrial functions such as metabolism.

In the Supplemental Material, the classical model of depression—the chronic unpredictable mild stress dataset (GSE151807)—was utilized to perform WGCNA with IL-33-overexpression mouse differential genes (Figure S1 of Supplementary Material), and the blue module was selected as being positively correlated with the control group and WT daytime traits and negatively correlated with the IL-33-overexpression group, model group, and night group. KEGG analysis of the genes combined in the blue module showed multiple metabolism-related pathways (Supplementary Figure S1F), including oxidative phosphorylation, inositol phosphate metabolism, carbon metabolism, fatty acid metabolism, glycolic acid metabolism, and glycosphingolipid biosynthesis-ganglio series. In addition to metabolic pathways, it also includes the following pathways (Supplementary Figure S1G): Huntington’s disease, Parkinson’s disease, pathways of neurodegeneration, amyotrophic lateral sclerosis, and prion disease. IL-33-overexpression, a model of CUMS (chronic unpredictable mild stress), and genes associated with circadian traits are enriched in pathways related to mitochondrial functions, such as metabolism, and are associated with Huntington’s disease, Parkinson’s disease, neurodegeneration pathways, amyotrophic lateral sclerosis, and prion diseases.

**Figure 5:**
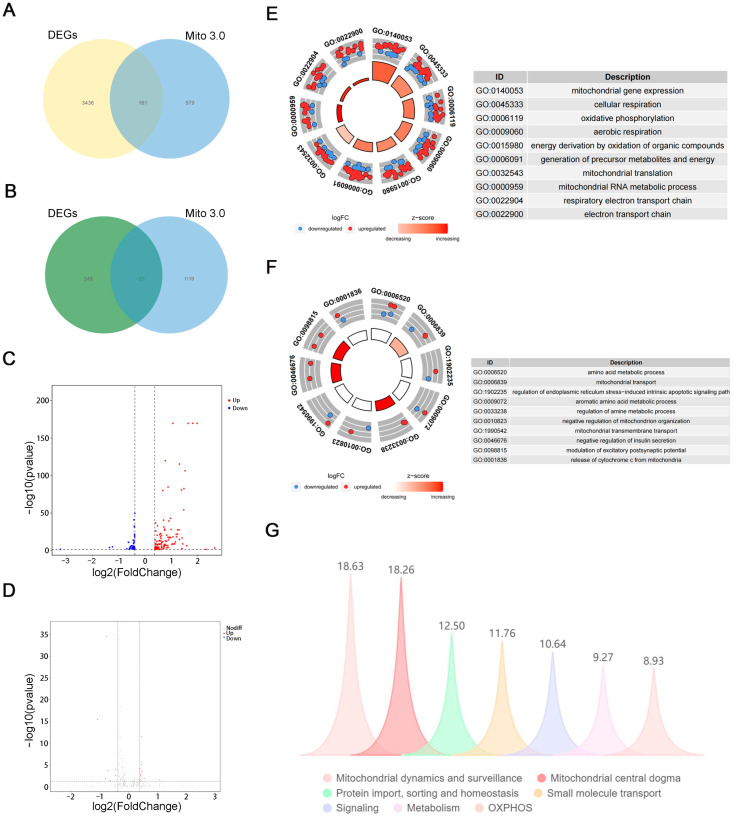
Mitochondrial pathways were enriched for differential genes in IL-33-overexpressing mice during light and dark periods. (**A**,**B**) Wayne plots of differential genes in IL-33-overexpressing mice interacting with genes in the Mito 3.0 dataset of the daytime and the nighttime, respectively. (**C**,**D**) Volcano plots of differentially expressed genes of the daytime and the nighttime, respectively. (**E**,**F**) GO analysis of differentially expressed genes in the daytime and in the nighttime. (**G**) The percentage of differentially expressed genes in mitochondria-associated pathways in the Mito dataset.

The intersection of differential genes of IL-33-overexpressing mice in the Day with the Mito 3.0 dataset yielded 161 genes related to mitochondria, and the intersection of differential genes at night of IL-33-overexpressing mice with the Mito 3.0 dataset yielded 21 genes related to mitochondria (Figure 5A,B). Results C and D of Figure 5 show that the differential genes were mainly concentrated in the light period and were predominantly increasing. GO analysis of daytime and nighttime differential genes showed that daytime genes were associated with mitochondrial gene expression, cellular respiration, oxidative phosphorylation, aerobic respiration, energy derivation by oxidation of organic compounds, generation of precursor metabolites and energy, mitochondrial translation, mitochondrial RNA metabolic process, respiratory electron transport chain, and electron transport chain (Figure 5E). The nighttime differential genes were associated with amino acid metabolic processes and mitochondrial transport (Figure 5F). Our Mito 3.0-associated mitochondrial pathway analyses of 161 daytime differential genes showed that the top three mitochondria-associated pathways with the highest percentage of differential base enrichment were mitochondrial dynamics and surveillance, mitochondrial central dogma, protein import, sorting, and homeostasis (Figure 5G).

### 2.5. QPCR

**Figure 6:**
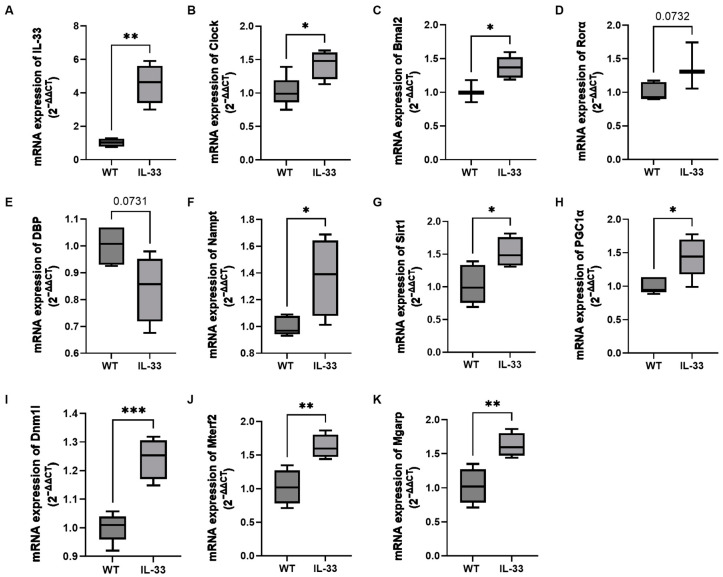
RT-qPCR results of some differential genes in IL-33-overexpressing mice during the daytime. (**A**–**K**) Statistical plots of the RT-qPCR results of *IL-33*, *Clock*, *Bmal2*, *Rora*, *DPB*, *Nampt*, *Sirt1*, *PGC1a*, *Dnm1l*, *Mterf2*, and *Mgarp* genes, respectively. There were 3–5 mice in each group, statistically different from WT mice (* *p* < 0.05, ** *p* < 0.01, and *** *p* < 0.001).

The results of RT-qPCR of some differential genes showed that *IL-33*, *Clock*, *Bmal2*, *Nampt*, *Sirt1*, *PGC1α*, *Dnm1l*, *Mterf2*, and *Mgarp* were upregulated in IL-33-overexpressing mice compared with WT mice (Figure 6). In addition, there was a trend of increased expression of *Rorα* and decreased expression of *Dbp*, which is in agreement with the transcriptomics results. Corresponding gene expression at night is shown in Supplementary Figure S2. The results of RT-qPCR were generally consistent with those of transcriptomics.

## 3. Discussion

The present study found that IL-33-overexpressing mice showed an 18.15% longer immobilization time in the FST and a 47.03% longer time in the TST compared to WT mice. In the SPT, IL-33-overexpressing mice had a 16.54% lower preference for sugar water than WT mice, suggesting they exhibit depression-like behavior. This finding is consistent with the results of previous studies showing that the IL-33 concentration in the cerebrospinal fluid was significantly associated with the development of perinatal depression [20], and that serum IL-33 levels were elevated in patients with major depression and depression combined with PTSD [21]. Another study found that treatment of major depression by transcranial direct current stimulation resulted in no significant change in plasma IL-33 levels before and after treatment [22]. Women with a history of recurrent major depressive disorder (rMDD) were found to have higher serum IL-33 levels in a more comprehensive cross-species study of humans and rats. Acute stress also increased IL-33 levels in the paraventricular nucleus of the hypothalamus and the prefrontal cortex in rats. These findings support the potential contributory role of IL-33 in the risk of MDD recurrence [23]. The alterations in IL-33 levels observed in the aforementioned studies were found to be inconsistent, likely attributable to variations in the causes of depression and different tissues analyzed (e.g., serum, cerebrospinal fluid, prefrontal cortex, hippocampus, and paraventricular nucleus of the hypothalamus). These findings suggest that depression is a complex disease that is associated with IL-33, although the mechanism requires further investigation.

We found changes in the circadian rhythm behavior of mice overexpressing IL-33. WT mice and IL-33-overexpressing mice exhibited a circadian rhythm in their daily activities, with a low number of activities during the light period (daytime) and an increased number of activities during the dark period (nighttime). Notably, IL-33-overexpressing mice exhibited more activity during the light period (9:00–18:00) compared to wild-type (WT) mice, while their activity levels during the dark period (21:00–6:00) were similar to those of the wild-type group. The trough and onset periods of the activity of IL-33-overexpressing mice were consistent with those of WT mice, but the peak period was one time period earlier than that of WT mice. It is suggested that the altered circadian rhythm activity is associated with depression-like behavior in IL-33-overexpressing mice. To gain a clearer understanding of the relationship between altered circadian rhythm and depression-like behavior in IL-33-overexpressing mice, we utilized a transcriptomic analysis to identify differential gene expression at different times of the day and night. The heatmap revealed that some genes are highly expressed during the day (red) but show reduced levels at night (blue) in WT mice. This presents a circadian rhythm in their mRNA expression.

Changes in mRNA expression rhythms in IL-33-overexpressing mice were clearly lost from the differential gene expression heatmap. Core clock genes exhibited differential mRNA expression primarily during the daytime (Table 1). Only BMAL2 mRNA levels increased at night, while no significant differences were found in other core clock genes when compared to wild-type (WT) mice. Under 12 h of light/12 h of darkness, IL-33-overexpressing mice exhibited peak activity time periods from 18:00 to 21:00, earlier than those observed in WT mice. In the course of running-wheel experiments, a shortening of circadian rhythms was observed in IL-33-overexpressing mice. This finding correlates with increased expression of Clock in the hippocampus, which has been shown to lengthen circadian rhythms after Clock gene mutation [24,25]. Analyses of clinical data on depression have found that clock genes are strongly associated with depression and may be potential therapeutic targets for depression [26]. Studies on IL-33 and circadian rhythm-related studies are limited. Additionally, the threshold for IL-33-dependent mast cell activation exhibits circadian variations [27]. IL-33 promotes mast cells to release IL-6, IL-13, TNFα, and other inflammatory factors. This process is dependent on the core clock gene Clock [28]. IL-33-overexpressing mice showed alterations in circadian rhythms, manifesting as increased activity during the sleep period, earlier peak activity, and shorter circadian rhythm cycles. Changes in transcriptional rhythms in IL-33-overexpressing mice may explain depression-like behaviors in IL-33-overexpressing mice. Genetic evidence indicates that higher IL-33 mRNA expression in hippocampal tissue is linked to depression-like behaviors.

In recent years, mitochondrial rhythms have been found to serve as an important regulatory point linking overall and organ rhythms and clock molecular rhythms [13]. Mitochondrial dysfunction has been found to exist in neuroinflammation and is impaired in patients with depression. The mRNA expression of subunits of the mitochondrial oxidative respiratory complex showed that mRNAs with increased expression in the daytime hippocampus included *mt-Nd1*, *mt-Nd2*, *mt-Nd4*, *mt-Nd4l*, *mt-Nd5*, *mt-Nd6*, and *Ndufb2* in Complex I; *mt-Cytb* in Complex III; *mt-Co1*, *mt-Co2*, and *mt-Co3* in Complex IV and *mt-Atp6* and *Atp5l* in Complex V. The majority of the genes displaying increased expression were mitochondria-encoded, whereas those with decreased expression were *Uqcrc1* in complex III, *Cox6a2* in complex IV, and *Atp5b* in complex V, all of which were nuclear-encoded subunits. At night, hippocampal transcription revealed that the subunits with increased expression were *Ndufab1* in complex I and *Cox6b2* in complex IV, suggesting that the rhythm of mitochondrial oxidative respiratory complex expression was disturbed after IL-33-overexpression. An increase in oxidative phosphorylation complexes within the hippocampal CA region was found to be part of the stress protection mechanism [29]. Thus, mitochondria respond to circadian rhythm disruption during microinflammation by increasing oxidative respiratory complex expression, potentially as a protective response to stress. Silencing the core clock gene ARNTL/BMAL1 can eliminate the circadian rhythm of mitochondrial respiration in cells, indicating that these rhythms are regulated by rhythmic genes. Pharmacological methods to inhibit mitochondrial oxidative phosphorylation or mtDNA depletion in cells exhibit dysregulation of clock gene expression [30], suggesting that mitochondria influence circadian rhythms through retrograde signaling pathways such as oxidative phosphorylation and mtDNA. Clock regulates mitochondrial dynamics and bioenergetics, including oxidative phosphorylation and ATP production [31]. The mitochondrial respiratory chain complex subunits were also found to have circadian oscillations, and peak protein expression was present at different time points [32]. This finding suggests the existence of a complex regulatory mechanism controlling the expression of these subunits. The functions of mitochondrial respiratory chain complex subunits during circadian rhythms are unclear. It has been shown that after inhibition of mitochondrial respiratory chain complex II with the toxin 3-nitroprussic acid (3-NP), mice exhibit severe circadian rhythm defects, disruption of sleep patterns, disruption of rhythmic behaviors, such as overactivity in the normal sleep phase, and disruption of the rhythmicity of clock protein PER2 expression [32]. Mitochondrial energy adaptation-related molecular alterations in the hippocampus of IL-33-overexpressing mice occurred mainly during the daytime, such as increased mRNA expression of *PGC1α*, *OPA1*, *TOMM70a*, and *Drp1*. In the microinflammatory state, mitochondrial responses change, including alterations in the transcriptional coactivator peroxisome proliferator-activated receptor-γ (PPARγ) coactivator 1α (PGC1α) and related transcription factors; mTOR and endoplasmic reticulum stress signaling; TOM70-dependent mitochondrial protein import; cristae remodeling factors, including mitochondrial contact sites and the cristae organizing system (MICOS) and OPA1; lipid remodeling; and assembly and metabolite-dependent regulation of respiratory complexes [33]. Disturbed mitochondrial-associated protein mRNA expression circadian rhythms are associated with IL-33-induced depression-like behavior. Because circadian mitochondrial rhythms are regulated by clock genes, which also affect the expression of rhythm genes and alter hippocampal rhythms, further study of mitochondrial circadian rhythms is important for depression-related research.

GO enrichment analysis of the differential genes showed that the BP pathways with the smallest *p*-values were nucleic acid metabolic process, heterocycle metabolic process, cellular aromatic compound metabolic process, nucleobase-containing compound metabolic process, and nucleobase-containing compound metabolic process, all of which are related to metabolism. KEGG enrichment analysis of differentially expressed genes identified immune-related pathways such as Herpes simplex virus 1 infection and neuroactive ligand–receptor interaction, as well as mood-related pathways like nicotine addiction, calcium signaling pathway, dopaminergic synapse, long-term potentiation, glutamatergic synapse, circadian rhythm, and circadian entrainment. The glutamate system is highly affected by stress, and chronic stress disrupts glutamate release, glutamate clearance from synapses, and glutamate receptor expression, with regional specificity toward these effects [34]. Several studies have found that enhanced glutamate release and glutamate receptor expression are associated with chronic stress, along with reduced glutamate clearance/metabolism in the hippocampus [35,36]. Our findings suggest that metabolic alterations in IL-33-overexpressing mice are coregulated by immune, emotional, and circadian pathways. These changes may contribute depression-like behaviors by altering the hippocampal glutamatergic system.

An analysis of differential genes and mitochondria-related pathways in the MitoCarta 3.0 database revealed that these genes are primarily involved in the following critical pathways: mitochondrial dynamics and monitoring, mitochondrial central law, protein input classification, and homeostasis. Recent studies highlight that mitochondrial fusion and fission processes are closely linked to circadian rhythms and are regulated by clock genes [15,37]. Circadian rhythms are lost in cells with disrupted mitochondria, partly due to the lack of oscillations in respiratory activity in cells with healthy mitochondria. Dynamin-related protein 1 (DRP1) is a key mediator of mitochondrial fission, and DRP1 is phosphorylated in a circadian manner; blocking DRP1 function impairs the core biological clock. PGC1α is thought to be a major regulator of mitochondrial biogenesis, fine-tuning, and maintaining mitochondrial homeostasis along with the mitochondrial quality control systems (fission/fusion and mitochondrial autophagy). The second pathway with the highest differential gene enrichment in the Mito 3.0 database is the mitochondrial central dogma, indicating that IL-33 regulates mitochondrial transcription and translation through *Terf2*. It should be noted that only mtDNA alterations at the transcriptional level were observed in this study, and the relationship between the mitochondria-related protein content, mitochondrial oxidative respiration and the observed circadian rhythm changes in mtDNA transcription remain to be further investigated.

A more detailed and in-depth analysis of the big data was performed by analyzing transcriptomics data with datasets from web-based databases using a variety of methods, combined with datasets related to mitochondria. The present study has revealed that IL-33 plays a regulatory role in circadian rhythms and is associated with depression-like behaviors. The underlying mechanism may be related to the regulation of mitochondrial metabolism and mitochondrial dynamics by IL-33, which provides novel insights into the pathogenesis of depression. However, we have not yet elucidated the causal relationship between disruption of mitochondrial rhythms and depressive-like behavior in the present study, which is an interesting direction for future research. We build on existing research and our data analysis, employing various approaches to verify our experimental results and integrate them with laboratory theories, facilitating the discovery of new mechanisms and theories. On the basis of analyzing the biological information and combining the experimental results, we will comprehensively explore the mechanism by which microinflammation leads to depression-like behaviors through disruption of the mitochondrial circadian rhythm. We could not explain whether the rhythmic and depressive-like behaviors induced by IL-33-overexpressing mice are more closely related to IL-33 intracellular kernel transcriptional regulation or extracellular inflammatory factor signaling, or the specific process. Furthermore, the effects of these mechanisms on glial cells and neurons in the hippocampus require further experimental validation and analysis.

Mitochondria may be the true integrative hub linking inflammatory-circadian-depressive-like behaviors. This study of circadian rhythms in mitochondria may provide a blueprint for the development of chronotherapy, and “mitochondrial medicine” should rely on the comprehensive study of mitochondria and related processes, leveraging any emerging knowledge from basic research and combining advanced big data analytics to analyze the pathophysiological processes of disease more effectively.

## 4. Materials and Methods

### 4.1. Animals

Experiments involving mice were performed in accordance with the National Institutes of Health Guide for the Care and Use of Laboratory Animals. Male C57BL/6J mice were purchased from Changchun Yisi Animal Laboratory Co. (Changchun, China); IL-33-overexpressing mice were kindly provided by Prof. Ying Sun from Capital Medical University (Beijing, China). The construction of IL33 transgenic mice is detailed in reference [38]. The animal experiments were reviewed and approved by the Committee for Animal Care in Research at Jilin University. Mice (8–10 weeks old) were maintained under standard conditions of temperature (22 ± 2 °C), humidity (60%), and light (12:12 h light/dark cycle) with lights on at 7:00 a.m. Food and water were freely available during the experimental period. This experiment has been approved by the Ethics Committee of Jilin University (202340).

### 4.2. Behavioral Analysis

#### Sucrose Preference Test (SPT)

The sucrose preference test (SPT) was conducted over a 24 h period. This test assesses anhedonia-like behavior, a classical symptom of depression. Each animal was allowed free access to two bottles of 1% sucrose solution for 48 h during training. After a 6 h food and water deprivation period, the animals were allowed to freely choose between two bottles for a 24 h test session as follows: one contained a 1% sucrose solution and the other contained water. To avoid any place preference, the position of the bottles was reversed at the midpoint of the 24 h test. Sucrose preference was calculated as follows: sucrose solution consumed/total liquid consumed × 100.

### 4.3. Light–Dark Box Test (LDB)

When given the option between a black and white box (light and dark boxes), rodents prefer to move in the dark environment, but their exploratory habits encourage them to explore the bright environments. However, the bright light stimulation of the box inhibits the animal’s exploratory activities in the box. Using the animal’s conflicting psychology, the anti-anxiety or anxiety-inducing effects of drugs on mice can be evaluated. The light and dark box (25 cm × 12 cm × 12 cm) has an open doorway (3.5 cm × 3.5 cm hole) for animals to pass through. At the beginning of the experiment, the mice were placed in a dark box, and the camera monitor above the dark box was turned on to record for 7 min. The number of times the mice penetrated the box, their residence time in the dark box, and their activity were recorded within the next 5 min. After recording, the experimental animals were placed back into their original cages, and the boxes were cleaned by wiping the walls and bottom with 75% alcohol to eliminate the impact of animal odor on subsequent experiments. After the experiment, the number of times the mice passed through the box within 5 min was counted, as was the time they remained in the open box.

### 4.4. Tail Suspension Test (TST)

The TST is a classic and rapid method for evaluating the efficacy of antidepressants and sedatives. The tail of the experimental animal was fixed, and its head was suspended downward. The animal struggles in this environment to escape the dilemma, but despite efforts, it becomes intermittently immobile, indicating a state of “behavioral despair”. A camera system was used to record for 7 min, and the mice were counted for 5 min of inactivity.

### 4.5. Forced Swimming Test (FST)

For the FST, the mice were placed in a transparent cylindrical container filled with clean water (high × diameter, 25 cm × 10 cm). The tank was filled with warm water (24 ± 1 °C), which was replaced after each FST session. The mouse was gently placed in the device, and its activity was immediately recorded by video for 6 min. The immobility time of mice in the last 4 min was used as an evaluation indicator for their depression-like behavior. An effective immobility time is defined as when a mouse floats and makes only slight movements to keep its head above the surface of water. The total mobility, swimming, and climbing durations were assessed. Mice were considered immovable when they made only those movements necessary to keep their heads above water. The FST sessions were continuously video recorded from the side of the tank. By observing and recording the despair state of experimental animals, it is possible to evaluate the depressive state of mice. After the experiment was completed, the mice were removed, dried, and returned to their original cages. When collecting data, we counted the immobility time of mice in the last 4 min. All recorded FST sessions were scored by a blinded experimenter.

After the 24 h SPT, other depression like behavior tests were conducted sequentially as follows: LDB, TST, and FST, between 9:00 and 12:00 in the morning, with a two-day interval between each test. Take the hippocampus two days after the last behavioral test. The behavior detection time should be consistent with the transcriptomics and RT-qPCR sample collection time.

### 4.6. Running Wheel Test

The running wheel test is a classic experiment used to detect circadian rhythms in mice. Before the test, mice were kept in a standard environment L:D (12 h:12 h). At the beginning of the experiment, the mice were placed in constant darkness for 24 days, and the Mouse Running Wheel System (Sansbio, SA103A, Nanjing, China) recorded the time of each group of mice in the spontaneous running wheel every 5 min and counted the change in the starting time point of the wheel in the 24-day period, reflecting the presence or absence of changes in the rhythmic cycle. The result could be used as an indicator of mice’s circadian rhythm.

### 4.7. Autonomous Activity Detection

The test time of the Autonomous Activity Logger (Sansbio, SA-YLS-1C) was set to 24 h, and eight time zones were set up, that is, the total number of activities in each 3 h period was recorded, and the number of activities in each time zone was displayed and saved in real time. The activity ratio was defined as the ratio of the number of activities in a time zone to the total number of activities in 24 h. The peak and trough periods were the periods with the highest and lowest number of activities in the entire 24 h period, respectively. The voluntary activity test responded to changes in circadian rhythm activity in mice.

Activity rhythm detection: Conduct formal testing one week after adapting to the environment. Autonomous activity detection, with each mouse tested for 24 h and returned to a normal environment for one week. Collected the hippocampus between 9:00 and 12:00. After the adaptation period of the running wheel test, the mice were tested under dark conditions for 24 days, and subsequent experiments, such as RNA extraction, were not performed on the mice after running wheel testing.

### 4.8. RNA Extraction and Transcriptomic Sequencing

There were three mice per group for transcriptomic sequencing. Total RNA was isolated from the hippocampus using TRIzol Reagent (Invitrogen Life Technologies, Waltham, MA, USA), after which its concentration, quality, and integrity were determined using a NanoDrop spectrophotometer (Thermo Scientific, Waltham, MA, USA). Three micrograms of RNA were used as the input material for the RNA sample preparations. To generate sequencing libraries, mRNA was first purified from total RNA using poly-T oligo-attached magnetic beads. Fragmentation was performed using divalent cations at elevated temperatures in an Illumina proprietary fragmentation buffer. First-strand cDNA was synthesized using random oligonucleotides and SuperScript II. Second-strand cDNA synthesis was performed using DNA Polymerase I and RNase H. The remaining overhangs were converted into blunt ends via exonuclease/polymerase activity, and the enzymes were removed. After adenylation of the 3′ ends of the DNA fragments, Illumina PE adapter oligonucleotides were ligated for hybridization. To select cDNA fragments of the preferred 400–500 bp in length, the library fragments were purified using the AMPure XP system (Beckman Colter, Beverly, CA, USA). DNA fragments with ligated adaptor molecules at both ends were selectively enriched using Illumina PCR Primer Cocktail in a 15-cycle PCR reaction. Products were purified (AMPure XP system, Waltham, MA, USA) and then quantified using the Agilent high-sensitivity DNA assay on a Bioanalyzer 2100 system (Agilent, Santa Clara, CA, USA). Subsequently, the sequencing library was sequenced on a NovaSeq 6000 platform (Illumina), Shanghai Personal Biotechnology Co., Ltd. (Shanghai, China).

### 4.9. Transcriptome Analysis Flow

1. Quality control

Samples were sequenced on the platform to obtain image files, which were transformed by the software of the sequencing platform. The original data in FASTQ format (raw data) are generated. Sequencing data contain several connectors and low-quality reads; thus, we use fastp (0.22.0) software to filter the sequencing data to obtain high-quality sequences (clean data) for further analysis.

2. Reads mapping

The reference genome and gene annotation files were downloaded from a genome website. The filtered reads were then mapped to the reference genome using HISAT2 (v2.1.0).

3. Expression analysis

We used HTSeq (v0.9.1) statistics to compare the read count values for each gene as the original expression of the gene and then used FPKM (fragments per kilo bases per million fragments)/TPM (transcripts per million) to standardize the expression.

4. Differential expression analysis

The difference in gene expression was analyzed using DESeq2 (v1.38.3) with the following screening conditions: expression difference multiple |log2FoldChange| > 1.3, significant *p*-value < 0.05. At the same time, we used the ComplexHeatmap (v2.16.0) software package to perform a bi-directional clustering analysis of the different genes in the samples. We generated a heatmap according to the expression level of the same gene in different samples and the expression patterns of different genes in the same sample using the Euclidean method to calculate the distance and complete linkage.

### 4.10. Method to Cluster

#### Enrichment Analysis

We mapped all genes to terms in the Gene Ontology database and calculated the number of DEGs in each term. We used ClusterProfiler (v4.6.0) to perform GO enrichment analysis on the differential genes (all DEGs/up DEGs/down DEGs), calculate *p*-values using the hypergeometric distribution method (standard of significant enrichment: *p*-value < 0.05), and find the GO term with significantly enriched differential genes to determine the main biological functions performed by differential genes. ClusterProfiler (v4.6.0) software was used to perform the enrichment analysis of the KEGG pathways of differential genes, focusing on the significant enrichment pathway with *p*-values < 0.05.

### 4.11. Weighted Gene Correlation Network Analysis (WGCNA) Network Construction and Module Identification

Expression profiling data GSE181285 were downloaded from the GEO database (https://www.ncbi.nlm.nih.gov (accessed on 18 February 2025)) and combined with wild-type (WT) and IL-33-overexpressing mouse transcriptomics datasets from this study to remove batch effects using the combat function of the SVA package of R software (4.3.3). The top 10,000 most highly expressed genes among those co-expressed in the two datasets were selected for WGCNA. Co-expression networks were constructed using the “WGCNA” package in R software. First, the soft threshold β was determined using the Pick Soft Threshold function in the “WGCNA” package, and a correlation coefficient R^2^ > 0.8 was used as a criterion to determine the soft threshold β. The appropriate soft threshold was calculated and then weighed, and the adjacency matrix of the expression spectrum was constructed. The adjacency matrix was then converted to a topological overlap matrix (TOM) according to the TOM overlap computation formula, and a threshold value of 0.25 was used to merge similar modules. Next, the relationship between each gene module and the sample phenotype was calculated, and Pearson correlation analysis was used to determine the correlation between the gene modules and the depressed mice and circadian rhythm scores to identify the gene modules that were most strongly correlated with both depression and circadian rhythms.

### 4.12. GO and KEGG Pathway Analyses of Key Module Genes

KEGG and GO pathway enrichment analysis was performed by the “clusterProfiler” software package (v4.6.0) to identify potential pathways regulated by key modular genes. Adjusted *p* < 0.05 was used as the critical criterion.

### 4.13. RT-qPCR Test

After completing all behavioral tests, the mice were decapitated after inhalation of excess carbon dioxide during the light period 9:00–12:00 or dark period 21:00–0:00, and the hippocampus was quickly removed. Total RNA was extracted from the hippocampus using TRIzol reagent (Invitrogen, USA), and cDNA was synthesized with random hexamers (LABLEAD, F0202, Beijing, China). RT-qPCR was performed using specific primers and SYBR Mix (LABLEAD, R0202, Beijing, China). The three-step amplification protocol consisted of 3 min at 95 °C followed by 40 cycles of 95 °C for 15 s, 60 °C for 30 s, and 72 °C for 30 s (BIO-RAD, 788BR06579, Hercules, CA, USA). RNA quantification of target genes was calculated using the delta–delta Ct method. The Ct values were normalized to those of β-actin. The primers for the genes are shown in Supplemental Table S1.

### 4.14. Statistics

GraphPad Prism 7.0 (GraphPad Prism Software) was used for the analysis. Data were submitted to a normal distribution test and expressed as the mean ± standard error of the mean (SEM). Comparative behaviors and gene expressions of wild and IL-33-overexpressing mice were performed using Student’s *t*-test. Differences were considered significant when *p* < 0.05.

## Figures and Tables

**Table 1:** Expression changes in major clock genes.

Description		Day (n = 3)			Night (n = 3)		
	Clock Genes	Regulation	FoldChange	Padj	Regulation	FoldChange	Padj
Basic Helix-Loop-Helix ARNT Like 1 (BMAL1)	ARNTL	Nodiff	1.031372698	0.719962763	Nodiff	1.057042079	0.863401368
Basic Helix-Loop-Helix ARNT Like 2 (BMAL2)	ARNTL2	Upregulation	1.956792157	2.95989 × 10^−8^	Upregulation	1.44459817	0.030686938
Clock Circadian Regulator	CLOCK	Upregulation	2.747879918	6.4576 × 10^−108^	Nodiff	0.924425039	0.195916097
Cryptochrome Circadian Regulator 1	CRY1	Downregulation	0.682569501	6.43815 × 10^−6^	Nodiff	0.96219436	0.945357646
Cryptochrome Circadian Regulator 2	CRY2	Nodiff	0.802135304	3.84084 × 10^−13^	Nodiff	0.939919501	0.444109684
Period Circadian Regulator 1	PER1	Nodiff	0.902908143	0.015264539	Nodiff	0.943491936	0.413562256
Period Circadian Regulator 2	PER2	Nodiff	0.827218297	0.002699877	Nodiff	0.853072565	0.002512735
Period Circadian Regulator 3	PER3	Nodiff	1.118463898	0.077805859	Nodiff	1.121648928	0.066344864
RAR Related Orphan Receptor A	RORA	Upregulation	2.384385473	3.58562 × 10^−38^	Nodiff	1.214980351	0.010333444
Nuclear Receptor Subfamily 1 Group D Member 1	NR1D1	Downregulation	0.705692673	3.07345 × 10^−19^	Nodiff	1.061435024	0.544933443
Neuronal PAS Domain Protein 2	NPAS2	Downregulation	0.756632427	5.58775 × 10^−5^	Nodiff	1.189135749	0.024280149
Casein Kinase 1 Delta	CSNK1D	Nodiff	0.857246539	1.04882 × 10^−7^	Nodiff	0.95875038	0.539067964
Casein Kinase 1 Epsilon	CSNK1E	Nodiff	0.905536296	0.041933273	Nodiff	1.031196812	0.840758836
Timeless Circadian Regulator	TIMELESS	Nodiff	1.070544503	0.810099071	Nodiff	1.057186247	0.982767663
D site albumin promoter-binding protein	DBP	Downregulation	−0.522679915	2.6601 × 10^−12^	Nodiff	0.171766033	0.075369819

**Table 2:** Expression changes of mitochondrial oxidative respiratory complex subunits.

		Day (n = 3)			Night (n = 3)		
	Genes	FoldChange	Padj	Regulation	FoldChange	Padj	Regulation
CI subunits	mt-Nd1	3.150251376	4.37 × 10^−170^	Upregulation	0.856203	0.195842815	Nodiff
	mt-Nd2	1.853113554	4.24 × 10^−85^	Upregulation	0.889883426	0.218977604	Nodiff
	mt-Nd4	3.999349234	0	Upregulation	0.956165	0.800404065	Nodiff
	mt-Nd4l	5.046868284	0.026956998	Upregulation	0.706909	0.739137062	Nodiff
	mt-Nd5	2.903795749	7.00 × 10^−107^	Upregulation	1.033054	0.931290146	Nodiff
	mt-Nd6	2.101866606	7.25 × 10^−19^	Upregulation	1.136864	0.688223548	Nodiff
	Ndufab1	1.197892222	0.103323999	Nodiff	1.30329201	0.031383398	Upregulation
	Ndufb2	1.375622256	0.000477668	Upregulation	1.000057056	1	Nodiff
CIII subunits	mt-Cytb	3.548372753	0	Upregulation	0.953009166	0.394629994	Nodiff
	Uqcrc1	0.763365601	2.14 × 10^−22^	Downregulation	0.917339254	0.104260544	Nodiff
CIV subunits	Cox6a2	0.393578215	0.000133101	Downregulation	0.79971873	0.503790598	Nodiff
	Cox6b2	1.241508988	0.550834996	Nodiff	2.110148254	0.025111541	Upregulation
	mt-Co1	2.103562125	0	Upregulation	0.978257093	0.880103489	Nodiff
	mt-Co2	2.804407948	7.78 × 10^−55^	Upregulation	0.875936502	0.406447032	Nodiff
	mt-Co3	1.694859453	1.34 × 10^−27^	Upregulation	0.937282381	0.757478171	Nodiff
CV subunits	Atp5b	0.768683702	3.11 × 10^−50^	Downregulation	0.87716178	2.83 × 10^−7^	Nodiff
	Atp5l	1.333111597	0.031152024	Upregulation	1.196845263	0.319040439	Nodiff
	mt-Atp6	2.49589186	3.43 × 10^−15^	Upregulation	0.804885115	0.196164846	Nodiff

## Data Availability

Data is contained within the article or Supplementary Materials.

## Supplementary Materials

The following supporting information can be downloaded at: https://www.mdpi.com/article/10.3390/ijms26051895/s1.
