# Supplementary material for: Changes in Mitochondrial Transcriptional Rhythms and Depression-like Behavior in the Hippocampus of IL-33-Overexpressing Mice
Source: Int J Mol Sci. 2025 Feb 22;26(5):1895. doi: 10.3390/ijms26051895 (PMC11900197; doi:10.3390/ijms26051895)
Supplement: Supplementary file 1 [file ijms-26-01895-s001.zip › Supplemental Figures S1 and S2.pdf]

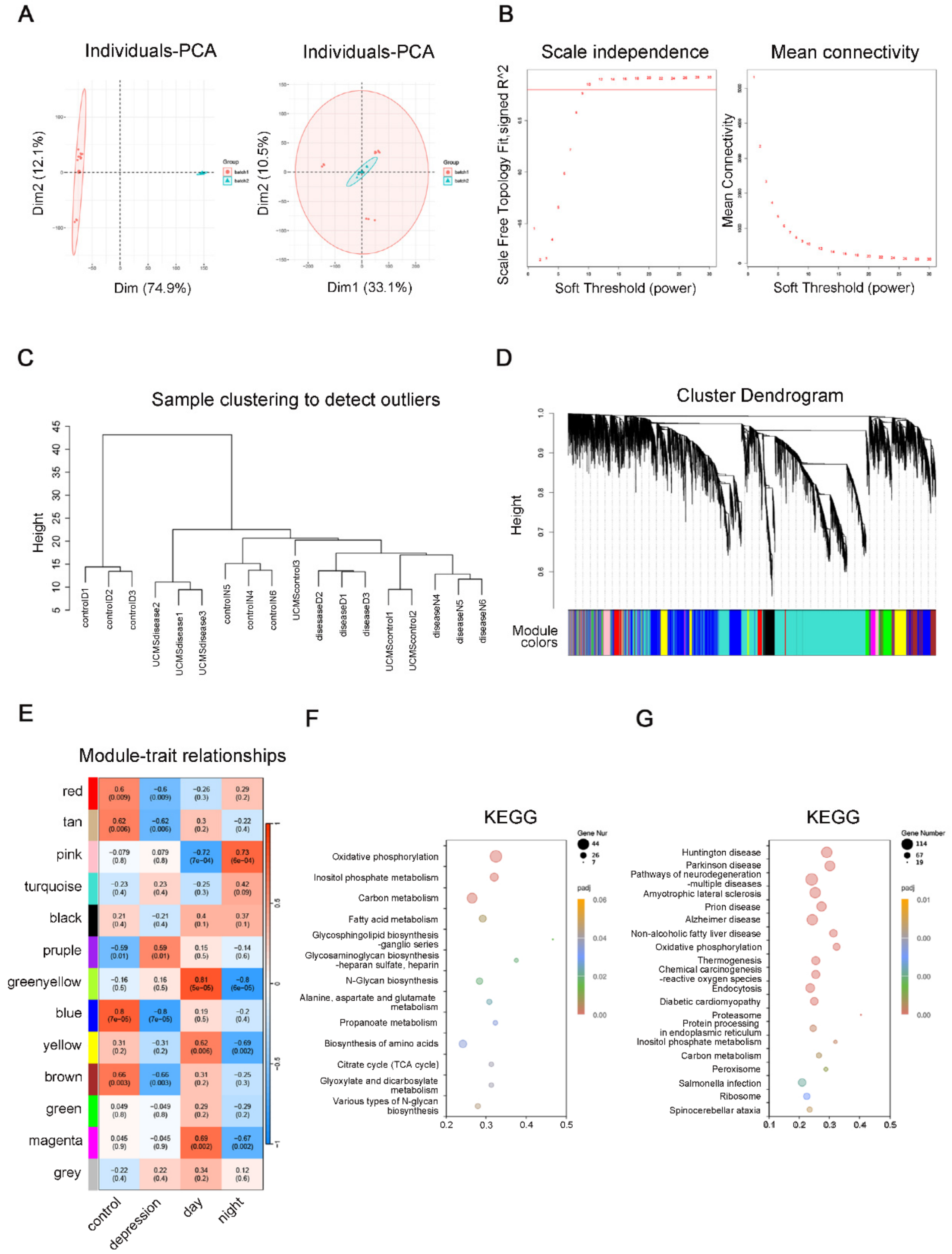

Supplementary Figure S1. WGCNA validation of differential genes in chronic unpredictable mild stress stimulation in mice and IL-33-overexpressing mice. (A) Before and after plots of the batch correction of the data. (B) Soft threshold set to 10 by the machine model. (C) Module plot. (D) Dendrogram. (E) Module-trait correlation analysis. (F) KEGG analysis results of the genes combined within the green and blue modules.

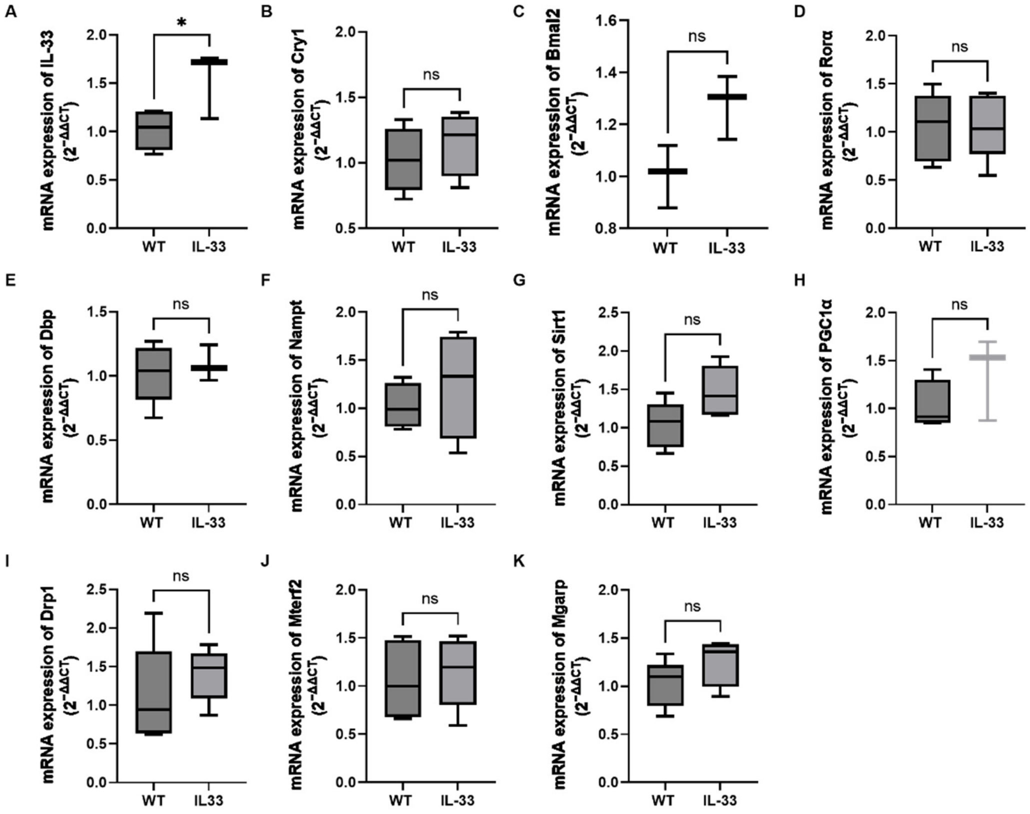

Supplementary Figure S2. RT-qPCR results of some differential genes at night in IL-33-overexpressing mice. A–K Statistical plots of the RT-qPCR results for *IL-33*, *Cry1*, *Bmal2*, *Rora*, *DPB, Nampt*, *Sirt1*, *PGC1α*, *Drp1*, *Mterf2*, and *Mgarp*, respectively. There were 3–5 mice in each group, statistically different from WT mice (* $p < 0.05$, ns means $p > 0.05$).
