# Supplementary material for: Changes in Mitochondrial Transcriptional Rhythms and Depression-like Behavior in the Hippocampus of IL-33-Overexpressing Mice
Source: Int J Mol Sci. 2025 Feb 22;26(5):1895. doi: 10.3390/ijms26051895 (PMC11900197; doi:10.3390/ijms26051895)
Supplement: Supplementary file 1 [file ijms-26-01895-s001.zip › Supplemental Table S1.pdf]

Supplemental Table S1. Primer sequences of major clock genes.

Primer Name	Squence
IL-33	5’-CTTCTCTGCCTATCCACGGG-3’
	5’-ATGTGTCAACAGACGCAGCA-3’
Clock	5’-ATGCCACAGAACAGTACCCA-3’
	5’-TTGTGTGGCGAAGGTAGGAT-3’
Bmal2	5’-CAGATGGAAAAGCGTCGGAG-3’
	5’-AGCTCTGTCATGCCTCTCAG-3’
Rorα	5’-GACGCCCACCTACAACATCT -3’
	5’-ACATATGGGTTCGGGTTTGA-3’
Dbp	5’-AAACAGCAAGCCCAAAGAACCG-3’
	5’-AAAAAGACTCGGGCCAGCCA-3’
Nampt	5’-ATTCAAGGAGATGGCGTGGA -3’
	5’- ACTTCTGTAGCAAAGCGCCA-3’
Sirt1	5’-CTCCTGTTGACCGATGGACT -3’
	5’-AGGATCGGTGCCAATCATGA -3’
Pgc1α	5’-TATGGAGTGACATAGAGTGTGCT -3’
	5’- CCACTTCAATCCACCCAGAAAG-3’
Dnm1l	5’- GCAACTGGAGAGGAATGCTG-3’
	5’- CACAATCTCGCTGTTCTCGG-3’
Mterf2	5’-AATGTCTTCAAGGGCAAGGC -3’
	5’- ACATAGAAGGGCTGGGCATT-3’
Mgarp	5’-CTGTGTCCAAGACTCTGGCG -3’
	5’-TTCCTGGATGACATTCGGCG -3’
β-actin	5’-AGCCATGTACGTAGCCATCC-3’
	5’-CTCTCAGCTGTGGTGGTGAA-3’
